# Role of 24-Hour Intraocular Pressure Monitoring in Glaucoma Management

**DOI:** 10.1155/2019/3632197

**Published:** 2019-09-19

**Authors:** Chun Hing Ho, Jasper K. W. Wong

**Affiliations:** ^1^Li Ka Shing Faculty of Medicine, The University of Hong Kong, Hong Kong, Hong Kong; ^2^Department of Ophthalmology, Li Ka Shing Faculty of Medicine, The University of Hong Kong, Hong Kong, Hong Kong

## Abstract

Glaucoma is the leading cause of irreversible blindness worldwide and the prevalence is on the rising trend. Intraocular pressure (IOP) reduction is the mainstay of treatment. The current practice of IOP monitoring is based on spot measurements during clinic visits during office hours. However, there are up to 50% of glaucoma patients who had normal initial IOP, while some treated patients continued to have progressive glaucomatous optic nerve damage even with a low IOP. Recent studies have shown that the IOP of glaucoma patients fluctuated during the day with different patterns, and some of them had peak IOP outside office hours. These findings provided us with new insights on the role of 24-hour IOP monitoring in managing normal tension glaucoma and patients with progressive deterioration despite apparently well-controlled IOP. Nevertheless, results to date are rather inconsistent, and there is no consensus yet. In this review, we briefly highlighted the current modalities of 24-hour IOP monitoring and summarized the characteristic 24-hour IOP pattern and the clinical relevance of IOP parameters in predicting glaucomatous progression in different glaucoma subtypes. We also discussed the therapeutic efficacy of current glaucoma treatment modalities with respect to the mentioned 24-hour IOP profiles, so as to strengthen the role of 24-hour IOP monitoring in identifying and stratifying the risks of progression in glaucoma patients, as well as optimizing treatments according to their IOP profiles.

## 1. Introduction

Glaucoma is defined as a group of progressive optic neuropathies characterized by the degeneration of retinal ganglion cells which results in optic nerve head changes and subsequent visual field loss [1, 2]. It is the leading cause of irreversible blindness worldwide [3]. The global prevalence of glaucoma in the population aged 40 to 80 years is 3.54%, and the number of people suffering from glaucoma is estimated to increase to 76.0 million in 2020 and 111.8 million in 2040 [4].

Elevated intraocular pressure is notably known to be a major risk factor for glaucoma progression [5] which is demonstrated by thinning in the retinal nerve fibre layer (RNFL) [6] and visual field deterioration [1, 2]. High intraocular pressure causes mechanical stress and strain on the posterior structures of eyes, particularly the lamina cribrosa, where the sclera is perforated and the retinal ganglion cell axons leave the eye [7]. Compression, deformation, and remodeling of the lamina cribrosa, which is the weakest point of the eye under intraocular pressure-induced stress, leads to disruption of axonal transport and neuronal damage in glaucoma [2, 8].

Interestingly, glaucomatous optic neuropathy can occur in individuals with a normal IOP, e.g., in normal tension glaucoma (NTG) [2, 9–11]. Glaucoma can also continue to progress in patients having satisfactory IOP control [11]. On the contrary, some individuals with high IOP may never develop any optic nerve damages, e.g., in ocular hypertension (OHT) [12]. Different mechanisms have been postulated to explain such phenomenon, including variations in optic nerve head (ONH) microcirculation [13–16] and pitfalls in IOP measurements.

Recent studies suggested that apart from spot office-hour IOP measurements [1], the peaks, rhythm, and fluctuations of IOP also had their roles in glaucoma progression [1]. In a study by Park et al. with juvenile open-angle glaucoma (JOAG) patients, although patients were on optimal medical treatment and presented with apparently well-controlled IOP, they still experienced temporary blurring of vision or progression of visual field defect. These patients were subsequently found to have wider diurnal IOP variations [11]. There is also evidence that more than half of the glaucoma patients have their peak IOP outside office hours [17]. A retrospective review also showed that the peak diurnal IOP was on average 4.9 mmHg higher than the maximum IOP conventionally measured in a clinic [17]. In a study comparing 24-hour IOP monitoring with the conventional office-hour IOP measurements, the implementation of 24-hour IOP monitoring even resulted in a change of clinical management in 79.3% of the patients [18]. These findings suggested that the current practice of IOP monitoring may indeed be inadequate and warrant further discussion [19].

In this review, we first briefly highlighted the current modalities of 24-hour IOP monitoring. We then summarized the 24-hour IOP patterns in different types of glaucoma and the correlation of different IOP parameters with glaucomatous progression. We will also present the evidence of the therapeutic efficacy of current glaucoma management modalities with respect to the 24-hour IOP profiles that we have analyzed.

## 2. Method

The PubMed database was searched from inception to 23 June 2019, with combinations of the following search terms: “glaucoma,” “intraocular pressure,” “diurnal,” “nocturnal,” “circadian,” “24-hour,” “monitoring,” “fluctuation,” “progression,” and “visual field” for randomized clinical trials, meta-analyses, systematic reviews, and interventional and observational studies. We included articles related to 24-hour intraocular pressure monitoring and therapeutic modalities in different glaucoma types based on extensive searches without language restrictions.

## 3. Modalities of 24-Hour IOP Monitoring in Clinical Practice

Common methods of intraocular pressure measurement in clinical practice include Goldmann applanation tonometry (GAT), handheld applanation tonometry, and pneumotonometry. These methods are mainly used for taking spot office IOP measurements but not for 24-hour IOP monitoring as these would be rather time consuming and inconvenient [20]. As IOP is dynamic in nature and is demonstrated to have diurnal changes and daily fluctuations, development of ambulatory 24-hour IOP measurement methods has been going on for the past several decades. These methods mainly include self-tonometry, permanent IOP monitoring, and temporary IOP monitoring.

Self-tonometry requires patients to do frequent and 24-hour sampling of their own IOP by using a handheld tonometer, e.g., Icare ONE rebound tonometry (RTONE). Sood and Ramanathan performed a study involving 18 patients with treated NTG using RTONE for 1 year [21]. The IOP measurements obtained with RTONE were shown to be strongly correlated with Goldmann applanation tonometer used in clinic visits, and for patients with NTG with a progression that was disproportionate to their office IOP measurements, the use of RTONE can reveal higher IOP spikes which was unidentified during office hours. Therefore, self-tonometry is definitely a valuable adjunct to spot office measurements as it provides a more complete data on the diurnal change of IOP which may assist in glaucoma management [22]. However, it should also be noted that self-tonometry is rather technically challenging for elder glaucoma patients and also could not take sleeping IOP measurements [23].

Permanent IOP monitoring involves surgical implantation of an IOP sensor, which is either a telemetric pressure transducer or a pressure sensor incorporated in an intraocular lens. Koutsonas et al. performed the first prospective clinical study of telemetric intraocular pressure monitoring (ARGOS study) and commented the IOP sensor to be well tolerated by all patients despite there may be early postoperative anterior chamber inflammation, mild to moderate pupillary distortion, and pigment dispersion after surgery [24]. Koutsonas et al. also successfully obtained repeated and automated 24-hour IOP measurements using the prototype noncontact reading system in another feasibility study involving 6 POAG patients [25] and reported no serious adverse events in a long-term follow-up safety report [26]. Permanent IOP monitoring with an implanted IOP sensor is generally more reliable, and 24-hour data (including data during sleep) could be collected over a longer period of time for disease monitoring and clinical studies. However, this requires a comparatively larger scope of surgery and leads to potential safety concerns [23].

One FDA-approved temporary continuous pressure monitoring system is the SENSIMED Triggerfish® disposable contact lens sensor (Triggerfish CLS, Sensimed AG, Lausanne, Switzerland). The satisfactory tolerability measured using the visual analogue scale, where 0 equals to no discomfort and 100 equals to very severe discomfort, ranged from 21.82 to 27.2 reported in a few studies [20, 27, 28]. The Triggerfish CLS is better than the aforementioned two methods in a sense that it is noninvasive and with easy reversibility and widespread availability [23]. It can also monitor IOP fluctuations for up to 24 hours including that during undisturbed sleep [23]. The Triggerfish CLS was also demonstrated to have a high reproducibility by Mansouri et al. [28], Mottet et al. [29], and Holló et al. [30]. However, the validity of CLS still remains unknown. This may be due to the fact that the CLS is not directly measuring IOP, but instead, this pressure monitoring system makes use of the measured curvature changes of the corneal limbus to estimate the variations in IOP [29, 30]. According to current studies, although the correlation of IOP measurements taken with GAT and CLS values was shown to be high at the time around CLS insertion [31], the correlation was shown to be poor after 24 hours of CLS insertion [29, 30]. This discrepancy might be explained by the observation that CLS curves showed a trend of increasing measurement values with an increasing time of CLS wear [30]. In addition, the CLS is also associated with common side effects including blurred vision, conjunctival hyperemia, and superficial punctate keratitis, which are generally considered to be mild and would resolve within 48 hours [28].

## 4. Characteristic 24-Hour IOP Patterns and Their Progression-Predicting Value in Different Types of Glaucoma

In normal individuals, the mean IOP is 14.7 ± 2.8 mmHg [32] and the short-term IOP fluctuation is measured to be around 4–6 mmHg [9]. Typically, IOP peaks in the morning and early afternoon and troughs in the afternoon and at night [6, 19, 33], and there might also be a spike of IOP upon waking [34].

Whereas, glaucoma in general is associated with higher spikes in 24-hour IOP monitoring which also takes longer time for dissipation [34]. Short-term IOP fluctuations are found to be significantly larger in glaucoma patients than in normal individuals, measuring 6–15 mmHg. Their IOP fluctuations could even go up to 40 mmHg in extreme cases [19, 35–37]. Diurnal IOP fluctuations range from 4.8 to 11 mmHg in glaucoma patients, compared to only 3.17–5 mmHg in normal individuals [38]. A study involving 296 eyes of 213 patients suffering from POAG and primary angle closure glaucoma (PACG) reported that a diurnal fluctuation larger than 6 mmHg with an IOP ≥ 21 mmHg would not be seen in normal eyes [39]. Diurnal and nocturnal IOP peaks were also found to be more frequent in glaucoma patients [40, 41].

A number of studies have tried to analyze the data from the Advanced Glaucoma Intervention Study (AGIS). Nouri-Mahdavi et al. [42] reported IOP fluctuation to be a significant risk factor for visual field progression. However, this study also included data obtained after therapy which may have contributed to the IOP fluctuation in patients with a progressed visual field [43]. Caprioli and Coleman then analyzed the data restricted to the period before visual field progression and excluded patients treated with surgeries. They found that IOP fluctuation was only significantly associated with visual field progression in the low mean IOP tertile, and they suggested that IOP fluctuation has a stronger influence on visual field progression only when the IOP is low; and the mean IOP would otherwise be the predominant risk factor when the IOP is high [43–46]. Other studies analyzing AGIS [1, 42] and Glaucoma Progression Study (GPS) [1, 40, 47] found that higher number of daytime peaks and long peaks [1, 48] and higher peak latency (shorter interval from peak to peak) were also associated with a faster rate of visual field decline. In contrast, some retrospective analyses of the Early Manifest Glaucoma Treatment Trial (EMGT) [49] and the Ocular Hypertension Treatment Study (OHTS) [50] did not find the association between IOP fluctuation with glaucomatous progression [51]. In addition, some other studies such as the European Glaucoma Prevention Study (EGPS), the Diagnostic Innovations in Glaucoma Study (DIGS) [52, 53], a study performed by Jonas and coworkers [19, 54], and a clinical study performed by Fogagnolo et al. [55] failed to show statistically significant influence of IOP fluctuation on glaucoma progression. However, some of the above studies, for example, OHTS did not obtain 24-hour IOP data and calculated the IOP fluctuation only as standard deviation of IOP at clinical visits [51]. However, in some study designs, patients were on different antiglaucoma medications which may alter the intrinsic glaucomatous IOP circadian rhythm. In consideration of the highly variable results from different glaucoma studies which include various glaucoma subtypes to further understand the role of 24-hour IOP patterns in glaucoma management, we highlighted the distinctive 24-hour IOP patterns and summarized the respective progression-predicting parameters in different types of glaucoma.

### 4.1. Primary and Secondary Open-Angle Glaucoma

Primary open-angle glaucoma (POAG) is the most common type of glaucoma [56]. The mean IOP was measured to be 19.9 ± 4.3 mmHg in a study involving 102 subjects using a noncontact tonometer every 2 hours starting from 8 a.m. to 12 a.m. [57], and was measured to be 17.6 ± 3.2 mmHg in another study involving 64 patients with POAG [58]. Both these findings were significantly higher than that in normal individuals which was only 14.7 ± 2.8 mmHg according to the Beijing Eye Study [32]. Regarding the 24-hour IOP pattern in POAG, IOP is highest in the morning [19, 59], gradually decreases over the day, and is lowest by midnight [17], sharing the same rhythm with normal individuals. A study by Xiao et al. showed that the peak IOP in 73.5% of the POAG group, as also in 59.6% of the normal control group, was outside working hours, typically during the time period from 12 a.m. to 6 a.m. [57], and the peak IOP could reach up to 25.3 ± 5.6 mmHg [57]. With such significant daily changes, short-term IOP fluctuation measured was much higher than that in normal subjects, ranging from 8.31 ± 2.58 mmHg [6, 19, 39] to 9.1 ± 3.6 mmHg [57]. In a retrospective review involving 29 treated POAG and NTG patients, Hughes et al. reported that the peak IOP during 24-hour monitoring could be almost 5 mmHg higher than the peak office measurements, and circadian measurements resulted in change in clinical management in up to 80% of patients [18, 33].

For secondary open-angle glaucoma such as pigmentary glaucoma and exfoliation glaucoma (XFG) in which the intermittent dispersion of pigments and exfoliation material into the trabecular meshwork is involved, their 24-hour IOP profiles are usually more variable. In a study involving patients suffering from pseudoexfoliation syndrome, Tojo et al. reported the mean IOP to be 20.3 ± 3.9 mmHg [60]. Konstas et al. found that the peak IOP in up to 45% of untreated exfoliation glaucoma was outside office hours [33, 36]. Jonas et al. reported that patients with secondary open-angle glaucoma had a pressure peak in the afternoon, compared to the morning peak in POAG and normal individuals [44, 61]. Secondary open-angle glaucoma also demonstrated a greater IOP variability compared with control and POAG patients [44, 61]. Patients with XFG typically exhibit a greater short-term IOP fluctuation, peak, and trough IOP than patients with POAG [19, 36, 62]. A clinical study by Konstas et al. showed a short-term IOP fluctuation higher than 15 mmHg in 35% of patients with XFG, but only in 7.5% of patients with POAG [19, 36].

The clinical importance of the 24-hour IOP pattern in POAG lies in its predictive value of disease progression. In a clinical study by Asrani et al., the respective hazard ratios of short-term and long-term IOP fluctuations for glaucomatous progression were 5.69 (95% CI, 1.86–17.35) and 5.76 (95% CI, 2.21–14.98) after adjusting for the office-hour IOP, age, race, gender, and visual field damage at baseline [58]. The correlation of short-term and long-term IOP fluctuation with visual field progression in POAG was also demonstrated in other studies of Asrani et al. [19, 33, 43, 44, 58], Naito et al. [63], Bergeå et al. [51, 64], and Rao et al. [65]. In a cross-sectional study conducted by De Moraes et al., the numbers of long peaks and mean peak ratio, which contribute to short-term IOP fluctuation, were also shown to be good predictors for glaucoma progression in POAG [1, 48]. For exfoliation glaucoma, Bergea et al. reported that both mean IOP and IOP fluctuations were correlated with visual field progression [43, 64]. The correlation of other IOP parameters with glaucomatous progression in POAG is however, not definite. In the study by Rao et al. involving 296 eyes of 213 POAG and PACG patients [65], the mean IOP and peak IOP were not found to be significantly correlated with the rate of visual field progression.

### 4.2. Normal Tension Glaucoma (NTG)

The mean IOP in NTG eyes ranged from 11.5 ± 2.4 mmHg [66] to 17.8 ± 1.6 mmHg according to Pajic et al. [67], similar to that in normal individuals. In a study involving 62 NTG patients, Quaranta et al. found that in the habitual position, 12.9% of patients exhibited diurnal acrophase, whereas 67.7% demonstrated nocturnal acrophase, differing from the typical rhythm of normal individuals [68]. Another study by Renard et al. also demonstrated diurnal (54.5%) or nocturnal (36.4%) [69] pressure spikes in NTG patients. Such nocturnal pressure spikes may then not be captured by office-hour IOP measurements [66, 70]. Nevertheless, diurnal IOP fluctuation in NTG patients was reported to be 4.4 ± 1.6 mmHg [6], which was not significantly different from normal individuals.

Choi et al. performed a hospital-based 24-hour IOP monitoring study involving 113 patients with untreated NTG, and a retrospective review in these NTG patients revealed that rapid glaucomatous progression was related to long-term IOP fluctuation in NTG [40, 51, 71]. A study including 140 patients with NTG also showed that those with significant long-term IOP fluctuation were 5.26 times more likely to demonstrate glaucoma progression [6], suggesting a strong relationship of long-term IOP fluctuation with glaucomatous progression in NTG. A retrospective chart review by Jonas et al. involving 855 eyes of 458 patients suggested that the mean IOP and the minimal IOP value were also independent predictors of glaucomatous progression among NTG patients [33, 54].

### 4.3. Primary Angle Closure Glaucoma (PACG)

In a retrospective analysis by Suresh et al., the mean IOP in PACG patients before treatment ranged from 29.9 mmHg to 45.8 mmHg [72]. The circadian IOP rhythm of PACG patients was similar to that of POAG patients, and normal individuals except those with PACG eyes had a consistently higher IOP [39]. As shown by a clinical study involving 53 PACG and 22 POAG patients, the mean trough IOP was higher in PACG patients (12.9 ± 2.8 mmHg) than POAG patients (11.5 ± 3.8 mmHg); and the mean midnight IOP level was also significantly higher in PACG (14.0 ± 3.2 mmHg) than in POAG patients (12.1 ± 3.7 mmHg) [17]. Short-term IOP fluctuation was also found to be higher in PACG patients (7.69 ± 3.03 mmHg) than normal individuals (4.83 ± 2.46 mmHg) in a study involving 75 eyes with PACG following iridotomy and 75 normal eyes as control [39].

While comparing groups of patients with primary angle closure suspect (PACS), primary angle closure (PAC), and primary angle closure glaucoma (PACG), the diurnal IOP fluctuation was significantly higher in PACG (5.4 ± 2.4 mmHg) and PAC (4.5 ± 2.3 mmHg) than in PACS subjects (3.7 ± 1.2 mmHg) and normal controls (3.8 ± 1.1 mmHg) [73]. Furthermore, in a study recording circadian IOP fluctuations by contact lens in 25 PACG eyes, short-term IOP fluctuations were found to be more prominent in patients with OCT and visual field evidence of progression, when compared to those who had stable disease [74]. Greater IOP fluctuation was also found to be associated with the extent of visual field pattern standard deviation (PSD) in PACG patients [73]. This evidence suggests that short-term IOP fluctuation correlates with the glaucomatous progression in patients with PACG.

### 4.4. Ocular Hypertension (OHT)

In a study conducted by Grippo et al. involving OHT patients, the mean IOP ranged from 21.1 mmHg to 24.2 mmHg [75]. Both sitting and supine IOP levels decreased progressively during the day and increased at night time. In the study, OHT patients had their IOP peaked at midnight, while the peak was in the early morning for glaucoma patients and control groups [75]. Concerning short-term IOP fluctuation, a study reported that it ranged from 6 mmHg to 8 mmHg, with a highest 15 mmHg possible in OHT [19, 35, 37, 76]. The diurnal IOP fluctuation was measured to be 6.8 mmHg in the OHT group, which was significantly higher than that of the healthy control group (5 mmHg) in another study analyzing the diurnal IOP curves of 690 patients [59].

Although the Early Manifest Glaucoma Trial, the Malmo Ocular Hypertension Study, the Ocular Hypertension Treatment Study, and some other studies failed to show statistically significant association between diurnal IOP fluctuation or long-term IOP fluctuation and glaucoma progression [34, 49, 53, 70, 77], they showed that long-term IOP fluctuation was higher in OHT patients who developed glaucoma (3.16 ± 1.35 mmHg) than those who did not convert (2.77 ± 1.11 mmHg) [53]. Those who progressed had more than 8 mmHg diurnal IOP fluctuation, compared to <6 mmHg in those who did not progress in certain other studies [78]. This might be explained by the fact that IOP fluctuations are positively correlated with the mean IOP and peak IOP [54], which are shown to be risk factors for conversion of OHT to POAG in multiple studies [19, 78, 79].

See Table 1 for the summary of 24-hour IOP patterns in normal individuals and different glaucoma types and the potential progression-predicting IOP parameters in different glaucoma types.

## 5. Therapeutic Efficacy of Current Glaucoma Management Modalities with respect to 24-Hour IOP Patterns

### 5.1. Medical Therapy

Medical therapy in glaucoma involves the use of prostaglandin analogues, beta-blockers, alpha-2 adrenergic agonists, parasympathomimetics, and carbonic anhydrase inhibitors commonly as topical eyedrops [82].

Prostaglandin analogues (travoprost, latanoprost, bimatoprost, tafluprost) are the most potent topical antiglaucoma agents which can achieve uniform 24-hour IOP reduction of 24–29% [83–86]. Stewart et al. conducted a meta-analysis of studies that assessed the 24-hour efficacy of antiglaucoma medications and concluded that bimatoprost followed by travoprost were the two most effective IOP-reducing medications [51, 85]. In patients with newly diagnosed NTG, topical travoprost (0.004%) was shown to significantly reduce mean IOP, maximum and minimum IOP, and short-term IOP fluctuations [87]. Seibold and Kahook found that travoprost significantly lowered IOP at all time points during diurnal and nocturnal periods in an interventional trial involving 27 NTG patients [88]. In a prospective crossover clinical trial involving 23 patients with POAG, Mansouri et al. found that prostaglandin analogues could flatten the increase of IOP at transition of the wake/sitting to sleep/supine period but did not have an effect on acrophase and amplitude [89]. In a crossover study by Orzalesi et al., it was reported that latanoprost had a superior efficacy in lowering IOP than timolol (beta-blocker) at multiple time points between 3 a.m. and 9 p.m. and at midnight and was also superior to dorzolamide (carbonic anhydrase inhibitor) at time points between 6 a.m. and 12 p.m., thus more potent and able to reduce IOP fluctuations [19, 83, 90]. Gil-Carrasco et al. also found that prostaglandin analogues were more effective than brinzolamide in reducing 24-hour mean IOP and IOP fluctuations in patients with POAG [91]. Prostaglandin analogues were also shown to be more effective than selective laser trabeculoplasty (SLT) in reducing 24-hour IOP fluctuations in POAG and NTG [70]. Notably, evening administration of latanoprost or the combination of latanoprost and timolol was able to lower daytime IOP than morning dosing in patients with POAG [19, 81, 83, 92]. A prospective placebo-controlled study by Konstas et al. also showed evening administration of tafluprost-timolol fixed combination having a higher 24-hour efficacy in optimizing the IOP profile in patients with POAG than morning administration [93]. In a controlled crossover trial involving 44 patients with POAG or OHT, the 24-hour efficacy of latanoprost, travoprost, and bimatoprost demonstrated no statistically significant difference, but some evidence suggested that travoprost and bimatoprost may offer a more uniform 24-hour IOP reduction than latanoprost [83, 85, 94–97].

Timolol 0.5% solution was shown in a meta-analysis to obtain a mean circadian IOP reduction of 19–24% from the untreated baseline despite reduced nocturnal efficacy [83, 85]. In a study involving 60 subjects with OAG or OHT by Seibold et al., timolol 0.5% solution was able to reduce IOP during the diurnal period, but did not lower IOP overnight [98]. Apart from timolol 0.5% solution, Quaranta et al. reported Timogel 0.1% (timolol 0.1% in gel-forming carbomers) to demonstrate a comparable efficacy with solution preparation to decrease mean 24-hour IOP, diurnal, nocturnal, and individual time point IOP [99]. The use of timolol in a carbomer gel form is able to achieve a similar control in the IOP profile, but in a lower concentration which reduces dry eye-like symptoms and make the medication better tolerated [100, 101]. Dorzolamide, when dosed three times daily, has been shown to lower circadian IOP by 15–23% [83, 85, 90, 102]. Orzalesi et al. also reported that timolol and dorzolamide shared similar efficacy, but dorzolamide exhibited significant superior nocturnal efficacy than timolol [83, 90]. Brimonidine (selective alpha-2 adrenergic agonist), when dosed twice daily, was also shown to reduce the mean 24-hour IOP by 17.3%, but demonstrated no nocturnal efficacy [83, 103]. Liu et al. found that as aqueous humour production has already decreased by 50% during sleep, aqueous suppressants (including a beta-blocker and selective alpha-2 adrenergic agonist) have little effect in lower IOP at night, explaining for the previously mentioned low nocturnal efficacy of both medications [43, 104, 105].

A meta-analysis investigated the effect of drug combinations in reduction of the mean diurnal IOP, and the relative reductions were found to be higher in combination of a beta-blocker and prostaglandin analogues (34.9% for travoprost/timolol, 34.3% for bimatoprost/timolol, and 33.9% for latanoprost/timolol fixed combinations) than the combination of a carbonic anhydrase inhibitor or alpha-2 agonist and beta-blocker (29.9% for dorzolamide/timolol and 28.1% for brimonidine/timolol combinations) [19, 106]. Latanoprost-timolol fixed combination could also decrease IOP more than latanoprost or timolol monotherapy at each time point of the 24-hour curve and decrease the level of IOP fluctuations [19, 107, 108].

### 5.2. Surgery

Common surgery techniques used in glaucoma include laser trabeculoplasty and trabeculectomy (also known as filtration surgery). Lee et al. evaluated the effect of laser trabeculoplasty on 28 eyes of 18 treated glaucoma patients and reported that laser treatment reduced IOP more consistently during the nocturnal period [51, 109], but it did not result in significant reduction in mean, peak, or diurnal IOP [33, 109, 110]. As previously mentioned, NTG patients typically exhibit either a diurnal or nocturnal IOP peaks [69], and 60% of the individuals have their peak during nocturnal hours [40]. Laser trabeculoplasty has been shown to increase outflow facility which is markedly reduced at night, resulting in a marked reduction of nocturnal IOP fluctuations [43], which may be important to prevent the progression of NTG although the range of 24-hour IOP fluctuations and diurnal IOP fluctuation does not change significantly after laser trabeculoplasty [111]. Also in patients with POAG eyes, Kóthy et al. has demonstrated that in a clinical trial involving 26 eyes of 13 patients the efficacy of selective laser trabeculoplasty is mainly efficacious in decreasing the amplitude of diurnal IOP fluctuation, but not in decreasing the mean IOP to a significant degree [112].

Trabeculectomy was particularly effective in reducing diurnal IOP fluctuation in POAG [15, 113] and PACG [114] patients and resulted in slower disease progression [34, 53, 114]. In a study by Medeiros et al., comparing the trabeculectomy and medical therapy in glaucoma patients, postoperative short-term IOP fluctuation was reduced to 2.3 ± 0.8 mmHg, whereas that in medically treated group is 4.8 ± 2.3 mmHg [19, 39, 115]. A prospective observational study by Mansouri et al. involving 60 patients showed that the mean diurnal IOP of the trabeculectomy treated patients was significantly lower than that of the patients who were treated with latanoprost monotherapy or deep sclerectomy with collagen implant [33, 110]. A study by Konstas et al. compared advanced OAG patients treated with trabeculectomy and patients on maximal medical therapy, and another study by Ross et al. also demonstrated that a successful trabeculectomy was more effective to achieve a lower mean IOP, peak, and fluctuations of IOP over 24 hours [33, 116, 117]. A case study of Park et al. also demonstrated a greater effectiveness of trabeculectomy in reducing diurnal IOP fluctuation than optimal medical treatment in cases of juvenile open-angle glaucoma [11].

See Table 2 for the summary of therapeutic efficacy of current glaucoma management modalities with respect to 24-hour IOP patterns.

## 6. Discussion

In the current clinical practice, patients' intraocular pressure profiles are mainly appreciated with spot office-hour IOP measurements using Goldmann applanation tonometry (GAT), handheld applanation tonometry, and pneumotonometry. In view of IOP monitoring and assessment of glaucomatous progression, interestingly there are individuals with normal IOP readings or satisfactory IOP control [2, 9–12] developing visual field deterioration and even irreversible blindness. Recent studies suggested that apart from spot office-hour IOP measurements, peaks, rhythm, and fluctuations of IOP also had their roles in glaucoma progression [1, 40, 42, 47, 48], but some studies suggested otherwise [19, 49, 50, 52–55]. We hypothesized that such controversy may be explained by the difference in study designs and the variety in glaucoma subtypes. Therefore, we reviewed the literature and characterized the important findings of the studies regarding the 24-hour IOP patterns of primary and secondary open-angle glaucoma, normal tension glaucoma (NTG), primary angle closure glaucoma (PACG), and ocular hypertension (OHT).

Mean IOP, which we may also be able to appreciate by averaging spot office IOP measurements, and short-term IOP fluctuations were reported to be higher than normal healthy individuals [6, 19, 32] in patients with POAG [6, 19, 39, 57, 58], secondary open-angle glaucoma (e.g., XFG) [19, 36, 60], PACG [39, 72], and OHT [19, 35, 37, 59, 75, 76], but not in patients with NTG [6, 66]. The circadian IOP rhythm in POAG was generally similar to normal individuals that the IOP level was highest in the morning and gradually decreased over the day [6, 17, 19, 33, 34, 59]. However, there were more frequent morning peaks in POAG and the peak IOP was also reported to be outside of working hours in 73.5% of patients [57]. In comparison to POAG, secondary open-angle glaucoma, e.g., XFG, demonstrated a different and more fluctuated circadian pattern [19, 36]. It was usually associated with pressure peaks in the afternoon [44, 61], and therefore, the peak IOP was also not always easily captured during working hours [33, 36]. As short- and long-term IOP fluctuations [19, 33, 43, 44, 51, 58, 63–65], number of long peaks [1, 48], and mean peak ratio [1, 48] were also shown to be the progression-predicting parameters in POAG and secondary open-angle glaucoma, in clinical scenarios in which patients have visual field deterioration but satisfactory spot office IOP readings, 24-hour IOP monitoring allows clinicians to identify the morning and afternoon peaks and IOP fluctuations which may suggest subsequent management modifications. In NTG patients, 24-hour IOP monitoring may particularly be of paramount importance as the mean IOP measured was generally similar to normal individuals that we would not have much clue for diagnosis and subsequent monitoring of the disease progress. NTG patients typically had a predilection of diurnal or nocturnal IOP spikes [68, 69] and up to 60% of them experienced peak IOP spikes at midnights [66, 70]. It should be noted that short-term [51, 71] and long-term IOP fluctuations [6, 80] and minimal IOP value [54, 68] in addition to the mean IOP [54, 68] were all shown to be progression-predicting parameters in NTG. These suggested that we may need to apply 24-hour IOP monitoring in patients with NTG to predict the progression risk, guide diagnosis, and individualized management. PACG had a similar 24-hour IOP rhythm as normal individuals [39], whereas OHT had an inverted circadian pattern that IOP decreased along the day and increased during nocturnal hours [75]. Short-term IOP fluctuations [73, 74, 78], long-term fluctuations [53], and peak IOP [54, 78, 79, 81] measured with 24-hour IOP monitoring in PACG and OHT may also be valuable as an adjunct to office measurements to predict optic nerve head damage and visual field loss, i.e., the conversion from primary angle closure suspect or ocular hypertension, to PACG or POAG, respectively.

In view of the suggestion of setting a more comprehensive management target regarding patients' 24-hour IOP profiles, we have also discussed the effectiveness of glaucomatous medications, laser trabeculoplasty, and trabeculectomy in optimizing various 24-hour IOP parameters. According to current data, prostaglandin analogues (especially bimatoprost and travoprost [51, 85]) were the most effective topical eyedrops to achieve a uniform 24-hour IOP reduction [83–86], and they were also effective in reducing the maximum and minimum IOP and short-term IOP fluctuations [87]. In particular, evening administration of prostaglandin analogues was shown to be better than morning dosing to lower daytime IOP in patients with POAG [19, 81, 83, 92]. Alpha-2 adrenergic agonists and beta-blockers shared similar efficacy in reducing IOP but were associated with a lower nocturnal efficacy [83, 85, 98, 103], whereas carbonic anhydrase inhibitors had superior nocturnal efficacy in comparison [43, 83]. Among all types of drug combinations, fixed combinations of prostaglandin analogues and beta-blockers were the most effective in optimizing the 24-hour IOP pattern [19, 106]. Clinicians are thus recommended to choose different antiglaucoma monotherapy or drug combinations according to the distinct circadian rhythm in each glaucomatous subtype or as reflected by the individual IOP profile. It should however be noted that some antiglaucoma medications, e.g., timolol [119] and apraclonidine [120, 121], were shown to be associated with tachyphylaxis upon long-term administration in a few studies, and their long-term clinical significance with respect to the 24-hour IOP patterns is to be further explored. Regarding surgical options, selective laser trabeculoplasty, albeit not demonstrated to modify the overall 24-hour IOP pattern, was able to decrease nocturnal IOP spikes [43] which may contribute to NTG progression [111]. However, it should be noted that in POAG and NTG patients, prostaglandin analogues were shown to be more effective than laser trabeculoplasty in reducing short-term IOP fluctuation which predicted disease progression [70]. But trabeculectomy was shown to be more effective than medical therapy [45, 68, 71, 74] in reducing diurnal IOP fluctuation in POAG [15, 113] and PACG [114] patients and slowed down disease progression [34, 53, 114].

To implement 24-hour IOP monitoring in clinical practice, currently available methods include self-tonometry using a handheld tonometer, permanent IOP monitoring using a surgically implanted IOP sensor, and a temporary IOP monitoring system, such as the SENSIMED Triggerfish® disposable contact lens sensor (CLS). The CLS is mostly recommended for its noninvasiveness, easy reversibility [23], widespread availability [23], high tolerability [20, 27, 28], and high reproducibility [28–30]. However, the validity (i.e., the IOP estimation accuracy) [29, 30] of the CLS beyond 24 hours of usage and the cost effectiveness of CLS in the management of glaucoma are yet to be explored. It was estimated by the United Kingdom's National Institute for Health and Care Excellence that each 24-hour use of the SENSIMED Triggerfish® CLS costs between $526 and $682 in addition to the cost of around $7310 for the reusable data recorder, cable, and software [20].

## 7. Conclusion

After reviewing the current literature, we concluded that 24-hour IOP monitoring might potentially be a valuable adjunct to spot office IOP measurements as it offers clinicians information on the overall circadian IOP rhythm, timing of IOP peaks, and magnitude of IOP fluctuations in different glaucoma subtypes and in different patients. Various IOP parameters, including mean IOP, short-term and long-term IOP fluctuations, and minimal and peak IOP values, were shown in multiple studies to be predictive of glaucomatous progression. These parameters may have important roles in the early detection and future diagnosis of glaucoma with normal IOP, i.e., NTG, and in disease monitoring in patients with visual field deterioration yet satisfactory spot office IOP readings. However, it should be highlighted that in spite of the seemingly promising clinical significance of 24-hour IOP monitoring, the available data from the current literature are still heterogeneous in terms of patient characteristics, inclusion criteria, 24-hour IOP monitoring methods, and study methods to support the clinical implementation of 24-hour IOP monitoring. Large-scale standardized prospective trials would be essential to better correlate the 24-hour IOP patterns with glaucoma progression in different glaucoma subtypes and evaluate the efficacy of different treatment modalities in optimizing IOP patterns as discussed. Validity, cost effectiveness, and side effect profiles of various modalities of 24-hour IOP monitoring should also be further explored. Otherwise, with further scientific and clinical evidence, clinicians are encouraged to provide management targeting the poorly controlled IOP parameters identified in patients with glaucomatous progression that is disproportionate to spot office IOP measurements.

## Figures and Tables

**Table 1:** Summary of 24-hour IOP patterns in normal individuals and different glaucoma types and the potential progression-predicting IOP parameters in different glaucoma types.

Patient groups	24-hour IOP rhythm	Mean IOP (mmHg)	Short-term IOPf (mmHg)	Potential progression-predicting parameters	Other IOP characteristics
Normal control	Spikes upon waking [34], peaks in the morning and early afternoon, troughs in the afternoon and at night [6, 19, 33]	14.7 ± 2.8 [32]	4–6 [9]	—	—

Primary open-angle glaucoma (POAG)	Highest in the morning [19, 59], gradually decreases over the day, and lowest by midnight [17] with more frequent morning peaks (esp. 12 a.m. to 6 a.m.) [57]	17.6 ± 3.2 [58]; 19.9 ± 4.3 [57]	8.31 ± 2.58 [6, 19, 39]; 9.1 ± 3.6 [57]	Short-term IOPf and long-term IOPf [19, 33, 43, 44, 51, 58, 63–65], number of long peaks [1, 48], mean peak ratio [1, 48]	Peak IOP in 73.5% of patients was outside working hours [57]

Secondary open-angle glaucoma (e.g. XFG)	Pressure peak in the afternoon [44, 61]	20.3 ± 3.9 [60]	Greater than POAG (>15 in 35% of XFG and 7.5% of POAG) [19, 36]	Mean IOP [43], short-term IOPf [43]	Peak IOP in 45% of untreated XFG was outside working hours [33, 36], XFG exhibits greater peak and trough IOP than POAG [19, 36, 62]

Normal tension glaucoma (NTG)	Either diurnal or nocturnal pressure spike [68, 69], peak IOP in nocturnal hours in more than half of patients [66, 70]	11.5 ± 2.4 [66]; 17.8 ± 1.6 [67]	4.4 ± 1.6 [6]	Short-term IOPf [51, 71], long-term IOPf [6, 80], mean IOP [54, 68], minimal IOP value [54, 68]	—

Primary angle closure glaucoma (PACG)	Highest in the morning, gradually decreases over the day, and lowest by midnight [39]	29.9–45.8 [72]	7.69 ± 3.03 (PACG following iridotomy) [39]	Short-term IOPf [73, 74]	Mean trough IOP and mean midnight IOP levels are higher in PACG than in POAG [17], degree of diurnal IOP fluctuation: PACG > PAC > PACS [73]

Ocular hypertension (OHT)	Decreases during the day and increases at night time with peaks at midnight [75]	21.1–24.2 [75]	6–8 [19, 35, 37, 76]; 6.8 [59]	Mean IOP [54, 78, 79, 81], peak IOP [54, 78, 79, 81], short-term IOPf [78], long-term IOPf [53]	—

IOPf: IOP fluctuation; XFG: exfoliation glaucoma.

**Table 2:** Summary of therapeutic efficacy of current glaucoma management modalities with respect to 24-hour IOP patterns.

Treatment modalities	Therapeutic efficacy with respect to 24-hour IOP patterns
Prostaglandin analogues (e.g., travoprost, latanoprost, bimatoprost, tafluprost)	(i) Achieve a uniform 24-hour IOP reduction of 24–29% [83–86]
	(ii) Reduce mean IOP, maximum and minimum IOP, and short-term IOPf in NTG [87]
	(iii) Flatten the IOP increase at the transition of wake/sitting to sleep/supine period in POAG [89]
	(iv) Evening administration was better than morning dosing to lower daytime IOP in POAG [19, 81, 83, 92]
	(v) More effective than a beta-blocker and carbonic anhydrase inhibitor to lower IOP at multiple time points and reduce mean IOP and IOP fluctuations [19, 83, 90, 91]
	(vi) Bimatoprost followed by travoprost were the two most effective IOP-reducing medications [51, 85]
	(vii) More effective than selective laser trabeculoplasty in reducing 24-hour IOPf in POAG and NTG [70]

Beta-blocker (e.g., timolol)	(i) Achieve a mean 24-hour IOP reduction of 19–24% [83, 85]
	(ii) Low nocturnal efficacy in reducing IOP [83, 85, 98]
	(iii) Timolol 0.5% solution and Timogel 0.1% demonstrated similar efficacy to decrease mean 24-hour IOP, diurnal, nocturnal, and individual time point IOP [99], and Timogel 0.1% was better tolerated [100, 101]

Carbonic anhydrase inhibitor (e.g., dorzolamide)	(i) Achieve a mean 24-hour IOP reduction of 15–23% [83, 85, 90, 102]
	(ii) Superior nocturnal efficacy when compared to timolol [83, 90]

Alpha-2 adrenergic agonist (e.g., brimonidine)	(i) Achieve a mean 24-hour IOP reduction of 17.3% [83, 103]
	(ii) Low nocturnal efficacy in reducing IOP [83, 103]

Drug combinations	(i) Combination of prostaglandin analogues and beta-blockers was better than the combination of a carbonic anhydrase inhibitor or alpha-2 agonist and beta-blocker in reducing mean diurnal IOP [19, 106]
	(ii) Fixed combination of prostaglandin analogues and beta-blockers can decrease IOP more than respective monotherapy [19, 107, 108]
	(iii) Evening administration of prostaglandin-timolol fixed combination had a superior 24-hour efficacy in obtaining a better 24-hour IOP control than morning dosing in patients with POAG [19, 81, 83, 92, 93]

Laser trabeculoplasty	(i) Effective in reducing IOP and IOPf during nocturnal period [33, 109, 110]
	(ii) Could not significantly reduce mean, peak, or diurnal IOP [68, 111]

Trabeculectomy	(i) Effective in reducing diurnal IOPf in POAG and PACG [15, 113, 114, 118]
	(ii) Achieve a lower short-term IOPf, mean diurnal IOP, IOP peak than medical therapy [45, 68, 71, 74]

IOPf: IOP fluctuation.

## Abbreviations

IOP: Intraocular pressure
IOPf: Intraocular pressure fluctuation
ONH: Optic nerve head
POAG: Primary open-angle glaucoma
JOAG: Juvenile open-angle glaucoma
NTG: Normal tension glaucoma
PACG: Primary angle closure glaucoma
XFG: Exfoliation glaucoma
OHT: Ocular hypertension
RNFL: Retinal nerve fibre layer
RGC: Retinal ganglion cell.
